# Our Contemporaries

**Published:** 1916-03

**Authors:** 


### OUR CONTEMPORARIES.

Vermont Medicine, the official organ of the Vt. Medical Society, begins its first volume with the January issue. In a sense, it succeeds, after about a year's intermission, the Vermont Medical Monthly, ably conducted for many years by a private expression of public spirit, and which was abandoned because of lack of professional support. An economic issue is presented.

The Illinois Medical Journal, the official organ of the state society, in its December issue, protests against the usurpation of the Cook Co. Hospital by a group of Chicago surgeons prominent in the American College of Surgeons; also against the increase in the fee for post graduate students from $5 a year to $5 a month. It seems to us that the College can scarcely be held accountable for the acts of individual fellows; certainly not until a protest has been formally presented for its action. The question of hospital control is an old ethical problem which can never be satisfactorily settled except in strict accordance with the general principle that anything supported by the public shall not be exploited for personal benefit.

The Journal of Advanced Therapeutics, beginning with the January issue, is under new management and the name and, to some degree, the scope, are changed, the new title being American Journal of Electrotherapeutics and Radiology. The editorial staff remains the same with Dr. Wm. Benham Snow at the head.

**Ether in Infectious Gangrene.** L. Ombredanne, Bull. et Mem. Soc. Chirurg. de Paris, opens the wound, removes bits of projectile and clothing, makes parallel incisions over crepitating areas, washes with ether and applies gauze soaked in ether. An impermeable covering is applied. Dressings are changed daily for three or four days. Good results are usually noted in 3-6 days.

